# The Intestinal Microbiome in Juvenile Idiopathic Arthritis—Results of a Single-Center Pilot Study from Poland

**DOI:** 10.3390/jcm14176038

**Published:** 2025-08-26

**Authors:** Justyna Roszkiewicz, Jakub Lach, Monika Baranowska, Dominik Strapagiel, Krystyna Wyka, Elżbieta Smolewska

**Affiliations:** 1 Department of Pediatric Cardiology and Rheumatology, Medical University of Lodz, 91-738 Lodz, Poland; elzbieta.smolewska@umed.lodz.pl; 2 Biobank Lab, Faculty of Biology and Environmental Protection, University of Lodz, 90-236 Lodz, Poland; 3 BBMRI.pl Consortium, 50-367 Wrocław, Poland; 4 Department of Pediatrics, Hematology and Oncology, Medical University of Lodz, 91-738 Lodz, Poland

**Keywords:** juvenile idiopathic arthritis, microbiome, zonulin

## Abstract

**Background**: Altered microbiome structures are perceived as one of the factors triggering the rise of autoimmune diseases, including juvenile idiopathic arthritis (JIA). Despite the extensive research conducted on rheumatoid arthritis (RA), data on microbiome compositions in pediatric populations are scarce and inconclusive. Moreover, no study has addressed this issue in Polish patients with rheumatic diseases. **Objectives**: The aim of our study was to compare diversity in the fecal microbiome and concentration of the intestinal permeability marker zonulin in patients with new-onset JIA and healthy subjects. **Materials and Methods**: 15 treatment- naive patients with JIA and 15 age- and sex-matched controls qualified for the study. Analyses of fecal microbiome structure were performed using 16SrRNA gene sequencing, while concentration of serum zonulin was established using the ELISA method. **Results**: We found no statistically significant difference in alpha (*p* = 0.92) and beta diversity (*p* = 0.57)in the fecal microbiome between JIA patents and healthy children. Additionally, analyses of relative abundances of phyla, families, and genera identified no differences. Zonulin concentration did not vary (*p* = 0.88) between the study and control groups. **Conclusions**: There is no statistically significant difference in fecal microbiome structure between new-onset JIA patients and healthy controls.

## 1. Introduction

Juvenile idiopathic arthritis (JIA) is an umbrella term describing a heterogeneous group of autoimmune-driven arthritis occurring in the pediatric population. It is defined as persistent arthritis lasting for a minimum of six weeks in patients under 16 years of age [1]. The main histopathological feature of JIA is the synovitis caused by uncontrolled proliferation of synoviocytes and infiltration of the sublining layer of the synovium by immunocompetent cells, ultimately leading to articular destruction and ankylosis [2].

Despite the significant progress in JIA treatment that has been achieved in recent years, it is still one of the leading causes of disabilities in children, affecting their physical function, mobility, and overall quality of life [3]. Moreover, the reported prevalence of JIA worldwide is increasing, making it one of the three most common autoimmune diseases occurring in childhood [4]. The global incidence of JIA is estimated at between 1 and 23 cases per 100,000 children [5], whereas recently published data from Poland estimated disease incidence ranging between 24.0 and 38.7 per 100,000 children [6].

JIA is considered to result from an interplay between genetically determined tendencies regarding autoimmunity and acquired environmental factors, although the exact disease etiology is still elusive [4]. Genetic predisposition to JIA is mainly linked to class II HLA molecules (HLA-DRB1, HLA-DPB1) [7], although recent advancements in molecular genetics and the introduction of genome-wide association studies may lead to the identification novel risk loci (RELA, EBF1) [8]. New insights into disease pathogenesis are also supported by large-scale register-based cohort studies, identifying dysbiosis related interventions, i.e., early-life antibiotic exposure [9] and caesarean section delivery, as environmental factors associated with disease risk [10].

There is also growing evidence for the influence of the gut microbiome, a community of microorganisms that is essential for intestinal immune homeostasis, on the occurrence of many autoimmune diseases. Notably, 16S rRNA sequencing of fecal samples from patients with various autoimmune conditions revealed a correlation between the microbiome structure and disease progression in patients not only with rheumatic diseases [11] but also with autoimmune thyroid diseases [12] and Crohn disease [13], among others. Moreover, as proven in animal studies, interventions targeting the gut microbiome may result in a decrease in overall disease inflammatory activity.

The microbiome structure may influence the development of immune system cells, the integrity of the intestinal mucosal barrier, and the differentiation of T cell subsets, leading to dysregulation of the immune system and, in consequence, the development of autoimmunity [9]. The impact of microbiome structure on immune cells is mainly mediated via short-cell fatty acids (SCFAs) secreted by microbes, which increase the protective function of the intestinal epithelium, contributing to increased mucus production and local IgA secretion, preventing the development of “leaky gut” syndrome [14]. Zonulin is the physiological modulator of intracellular tight junctions, of which increased secretion (triggered by gut dysbiosis) leads to microbiota antigen and endotoxin trafficking from the intestinal lumen to the lamina propria, exacerbating innate and adaptive immune responses. These processes lead to excessive production of pro-inflammatory cytokines, such as tumor necrosis factor alpha (TNF-α) and interferon gamma (IFN-γ), in turn leading to a further opening of the paracellular pathway for antigens. Ultimately, this may break immune tolerance and trigger the onset of autoimmune diseases [15,16].

The impact of microbiome structure on autoimmune diseases has been described in other rheumatic conditions, especially in rheumatoid arthritis (RA), where numerous studies have described altered microbiomes of the lungs (reduced *Actinomycetaceae* and *Spirochaetaceae* in BAL samples), oral cavity (*Porphyromonas gingivalis, Prevotella*), and intestines (*Prevotella copri*, *Lactobacillus*, *Faecalibacterium*, *Streptococcus*) [17]. Based on our knowledge and a literature review, there are currently six studies focusing on the fecal microbiome of new-onset JIA patients and two describing the oral microbiome of the same patients.

Therefore, pediatric microbiome research in JIA is promising but still scarce. Existing studies point to dysbiosis (a loss of SCFA producers, reduced diversity in some cohorts) and subtype/geography-specific signatures, whereas adult RA has stronger evidence implicating specific taxa [18,19,20]. Collectively, these findings suggest that associations between microbiome composition and JIA may differ across specific patient populations and disease subtypes. Nevertheless, none of the studies has addressed the microbiome composition of Polish patients with RA or JIA. Therefore, the aim of our study was to compare the structure of the fecal microbiome composition and the serum concentration of zonulin, a tight-junction permeability marker, in Polish patients with JIA and healthy children.

## 2. Materials & Methods

### 2.1. Study Group Composition

Fifteen treatment-naive patients below the age of 16 with JIA (of all subtypes excluding systemic-onset JIA at the moment of disease diagnosis) and 15 healthy age-and-sex-matched controls qualified for the study. Diagnosis of JIA was based on ILAR criteria [1]. All patients were of Caucasian origin and from the same geographical location.

Children with other inflammatory conditions, infections, on elimination diets, or receiving antibiotic treatment or probiotic supplementation within three months prior to the study qualification visit were excluded from both the study and control groups. All the procedures conducted in this study were in accordance with the Declaration of Helsinki. Local Bioethical Committee approval (number RNN/243/19/KE) was obtained before study initiation, and informed written consent was acquired from patients’ legal representatives prior to study enrolment.

### 2.2. Laboratory Analysis

All patients enrolled in the study underwent standard biochemical assessment including measurements of inflammatory markers (C-reactive protein (CRP) and erythrocyte sendimentation rate (ESR)). Serum and fecal samples were collected at the baseline of the study and stored at −80°C until further analysis. The analysis of serum zonulin concentration was performed using a standard ELISA method according to the protocol of the manufacturer (Immundiagnostik kit K5601, Bensheim, Germany). DNA was extracted from the fecal samples using QIAamp DNA Stool Mini Kit (Qiagen, Hilden, Germany), according to the manufacturers’ protocols. Libraries were prepared following a two-step tailed PCR amplicon procedure based on the 16S Metagenomic Sequencing Library Preparation Protocol, as recommended by Illumina (Illumina Inc., San Diego, USA). The universal primer pair 16S-F and 16S-R was used to amplify the V3 and V4 of the 16S rRNA gene. The primers were modified to include Illumina™ overhang adaptors. All PCR plates included negative controls (no template) in which no contamination was detected for any of the negative samples. Sequencing adapters and sample-specific indexes were added to each amplicon via a second round of PCR using the Nextera XT Index kit (Illumina, Illumina Inc., San Diego, CA, USA). The quality of the DNA libraries was checked using High Sensitivity D1000Assay for 4200 Tape Station System (Agilent, Santa Clara, CA, USA). Quantitation of next-generation sequencing (NGS) libraries was done using NEBNext^®^ Library Quant Kit for Illumina^®^(New England Biolabs, Ipswich, MA, USA). Sequencing was performed on a pair-end setup on the Illumina Miseq Platform, yielding 2 × 250 bp pair-end sequences.

### 2.3. Data and Statistical Analyses

The distribution of normality for description data and zonulin concentrations was assessed using Shapiro-Wilk test. In independent groups, student’s *t*-test was used for comparisons of variables with normal distribution and Mann Whitney U-test for variables with non-normal distribution. Statistical analyses were performed using Statistica, version 13.3 (TIBCO Software Inc., Palo Alto, CA, USA). Sequencing data quality were analyzed using FastQC Adaptors [21], and low-quality sequences were removed from the reads with Trim Galore v. 0.6.4 [22] with default parameters. Further analysis was performed with QIIME 2 2019.10 [23]. DADA2 was used for denoising data and amplicon sequence variant (ASV) table generation. Both forward and reverse reads were trimmed at nucleotide 15 on the 5′ side and at nucleotides 245 and 239 on the 3′ side. Shannon index and Bray-Curtis distance were generated with the core-metrics-phylogenetic plugin with a sampling depth of 12,650, i.e., the number of reads that all samples were downsampled by to normalize them and reduce biases associated with differences in sequencing depths between individual samples. Alpha diversity comparisons were performed using the “diversity alpha-group-significance” plugin with the Kruskal–Wallis test for both “all groups” and “pairwise” tests. For beta diversity comparisons, the “diversity beta-group-significance” plugin with the PERMANOVA test was used. Taxonomic classification was performed using the “feature-classifier classify_sklearn” plugin based on the pre-formatted SILVA reference sequence and taxonomy files from QIIME 2 data resources. Analysis of composition of microbiomes (ANCOM) [24] was used to identify features that differed in abundance between groups.

## 3. Results

Both the study and control groups were composed of 15 patients: 14 girls (93.33%) and one boy (6.66%), with a mean age of 7.39 (±1.08, min. 2.01, max. 14.48) years in the study and 7.02 (±1.34, min. 2.28, max. 14.04) years in the control group (*p* = 0.78, student’s *t*-test). From the study group, 11 patients had been diagnosed with oligoarthritis (73.33%),two with polyarticular arthritis, of which one (6.66%) with the seropositive and one (6.66%) with the seronegative subtype, one (6.66%) with enthesitis-related arthritis (ERA), and one with psoriatic arthritis (6.66%). The mean number of inflamed joints in the study group was 2.26 (±0.53. min. 1.0, max. 8.0). The majority of patients (12/15, 80%) were ANA positive, and 30% had the HLA B27 antigen. The mean values of inflammatory markers were increased in JIA children, with a mean CRP value of 18.11 mg/L (±5.98, min. 0.7, max. 62.6; with a normal range < 5.0 mg/L) and 33.43 mm/h for ESR (±5.78, min. 8.0 max. 90.0; with a normal range < 15 mm/h), in contrast to healthy controls (mean CRP 1.78 mg/L ± 0.45, min. 0.2 max. 6.5), mean ESR 5.93 mm/h (±0.76, min. 2.0,max. 10.0; *p* values 0.01 and <0.01 respectively, student’s *t*-test) [Table 1].

**Figure 1:**
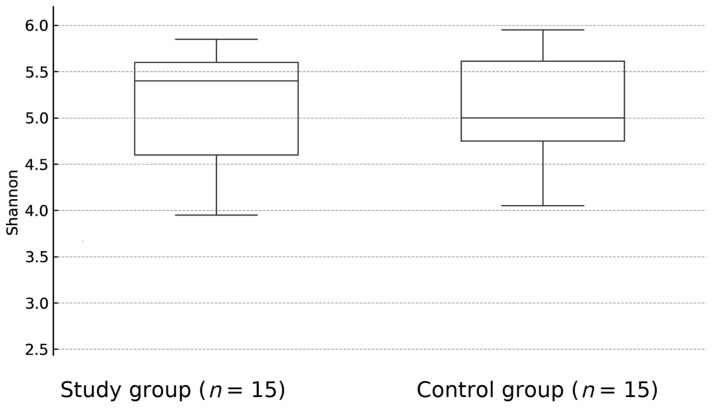
The difference between the overall biological diversity of patients with JIA and healthy controls fecal samples, expressed as Shannon index (*p* = 0.92).

**Figure 2:**
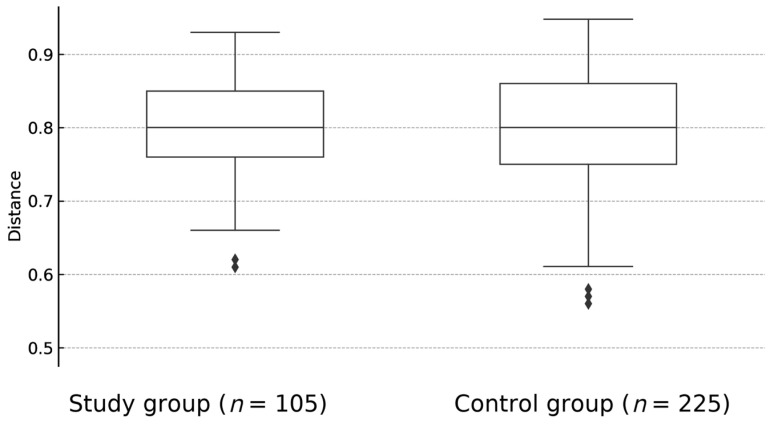
The difference between overall biological diversity of fecal samples of children with JIA and healthy controls, expressed as Bray-Curtis dissimilarity (*p* = 0.57). BC—Bray-Curtis.

There was no statistically significant difference between overall biological diversity—described as Shannon index (*p* = 0.92) (Figure 1) and Bray-Curtis distance (*p* = 0.57)—(Figure 2) between study and control groups. At the taxonomic level, no statistically significant differences in the microbiome composition were observed, although the proportion of *Firmictutes* to *Bacteroidetes* was shifted toward *Firmicutes* in both subgroups.

**Figure 3:**
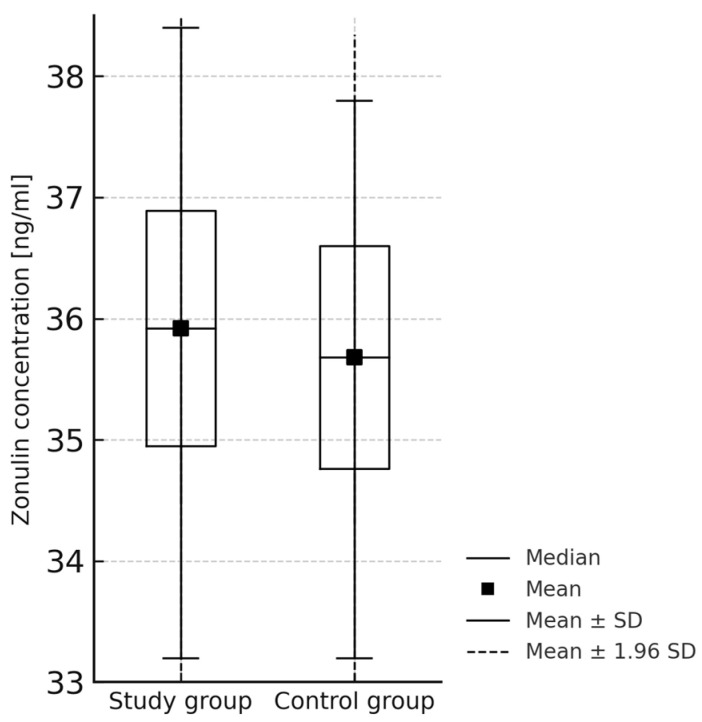
The difference between serum zonulin concentration between children with JIA (study group) and healthy controls. (Mann—Whitney U-test, *p* = 0.88).

Moreover, the mean serum zonulin concentration did not differ significantly between the study group and healthy controls, equalling 35.92 ng/mL (±1.44, min. 33.2, max. 38.4) and 35.68 ng/mL (±1.36, min. 33.2 max. 37.8), respectively. (*p* = 0.88, Mann—Whitney U-test) (Figure 3).

## 4. Discussion

In our study, we found no evidence for the microbiome malstructure in the pathogenesis of JIA. Despite the fact that JIA is the most common form of persistent arthritis in the pediatric population, the word “idiopathic” reflects our current ignorance regarding the exact disease pathogenesis. JIA, like other autoimmune diseases, is considered to be the result of an interplay between genetic and environmental factors. As genetic studies, including genome-wide association studies, have only partially explained the etiology of this complex disease [25], environmental factors are becoming the focus of extensive research. Recent advances in nucleotide sequencing technologies have led to the elucidation of the composition of the human microbiota and complex host–immune cell–microbiome interactions. The impact of microbiome composition on immune dysfunction in the course of RA has been well established, and *Porphyromonas gingivalis*, *Mycobacterium tuberculosis*, *Mycoplasma* spp., and *Proteus mirabilis* have been identified as microorganisms associated with the risk of developing this disease [17].

Several studies have addressed the issue of microbiome composition in JIA but have failed to establish a definite link. The heterogeneity of JIA, composed as it is of many subtypes with different clinical presentations and diverse immunological phenotypes, may be one of the factors associated with the lack of a universal pattern of microbiome disturbances in this group of patients. The most common findings in relevant studies include reduced microbial richness, relative abundance of *Bacteroides* [19,20], and a decreased bacterial α-diversity in children with JIA [18], although some studies did not reveal this association [26].

Multicenter studies conducted in different countries have shown that microbial structure alterations may be limited to particular populations, and the microbiome structure may remain altered during treatment and after achieving inactive disease stage. In a study conducted by Van Dijkhuizen et al., samples collected from Italian patients showed reduced microbial richness in comparison to healthy controls (*p* < 0.01), with a relative abundance of *Erysipelotrichaceae* and *Faecalibacterium prausnitzii*. Nevertheless, none of those differences were noted in the Dutch cohort [27].

In our study, we did not identify any statistically significant difference in microbiome composition between new-onset JIA and healthy children, as measured by the Shannon index, Bray-Curtis distance, and phylum level composition. Despite the sample size, our findings are comparable to those achieved by a multicenter Swedish study conducted by Öman et al. [28], which found no difference in alpha and beta diversity or relative abundance of taxa between treatment-naive JIA patients and healthy controls (including patients’ siblings).

Moreover, the majority of patients in our cohort had the most frequent JIA subtype oligoarthritis, and patients with systemic-onset JIA were excluded from the study due to a different disease etiology. In our research, the *Bacteroidetes*/*Firmicutes* ratio was shifted toward *Firmicutes* in both the study and control groups, which may have been the result of the different microbial structure of the Polish population. In our control group, the *Firmicutes*/*Bacteroidetes* ratio was 4.1, whereas in systematic reviews of the European population, this ratio was calculated as 3.21 [29]. Our study was enriched by a comparison of the concentrations of zonulin, an intestinal tight-junction permeability marker; it was found that the concentrations were comparable in both subgroups, which further supports our findings. None of the previous studies conducted with JIA patients has assessed zonulin concentration, although research conducted on adult patients with rheumatic conditions has revealed elevated zonulin levels at the early stages of the inflammatory process [30]; however, data about zonulin concentrations in established RA remain inconclusive. While a cross-sectional study by Mucientes et al. [31] found significant correlations between serum zonulin concentrations and TNF-α concentration in patients with rheumatoid arthritis (r = 0.266; *p* < 0.05), a study evaluating intestinal barrier markers in RA patients before and after DMARD treatment found that zonulin-related proteins did not differ significantly from controls and showed no change following DMARD therapy, suggesting that zonulin may be a less reliable barrier marker in well-established disease than in the preclinical stage [30].

While the gut microbiome is likely involved in the pathogenesis of JIA, there is currently no single, reproducible dysbiotic signature that can be considered a hallmark of the disease. The heterogeneity of existing studies and of JIA itself, the variation in microbial compositions across different populations, and the limited interventional evidence suggest that an altered microbiome structure should be regarded as a possible contributing factor rather than a defining or universal feature of JIA [20].

## 5. Conclusions

Although future studies are needed to verify our findings, our data and literature review suggest that the altered microbiome structure may not play a central role in the pathogenesis of JIA. Due to the low number of patients included in our study, our results should be interpreted as preliminary. Future studies involving a larger number of JIA patients are encouraged.

## Figures and Tables

**Table 1:** Basic demographic, clinical, and laboratory parameters of the study and control groups. Results are presented as % or mean± SD. ANA were considered positive if present at the titer at a minimum of 1:320.

Parameter	Study Group	Control Group	*p* Value
Age (years)	7.39 ± 1.08	7.02 ± 1.34	0.78
Sex			
Female (%)	93.33	93.33	1.0
Male (%)	6.66	6.66	
CRP (mg/L)	18.11 ± 5.98	1.78 ±0.45	0.01
ESR (mm/h)	33.43 ± 5.78	5.93 ±0.76	<0.01
Disease subtype (%)			
Oligoarthritis	73.33	n/a	-
Poliarticular seropositive	6.66		
Poliarticular seronegative	6.66		
Enthesitis-related arthritis	6.66		
Psoriatic arthritis	6.66		
Number of joints with active arthritis	2.26 ± 0.53	n/a	-
ANA positivity (%)	80	n/a	-
HLA B27 positivity (20%)	30	n/a	-

CRP—C-reactive protein, ESR—erythrocyte sedimentation rate, ANA—antinuclear antibodies, HLA—human leukocyte antigen, n/a—not applicable.

## Data Availability

All data relevant to the study are included in the article.
